# Gastric Irritation Producing Symptoms Resembling Apoplexy and Epilepsy

**Published:** 1830-01-01

**Authors:** 


### XLIV.

### Two Cases of Gastric Irritation producing Symptoms resembling Apoplexy and Epilepsy. By Professor Wood, of Philadelphia.*

A partial suspension of the cerebral functions is well known to be an occasional attendant upon irritation in the stomach. In some instances the symptoms so closely resemble those of diseased brain that the physician is in some danger of mistaking the real seat of the disorder, and consequently of misapplying his remedies. We may, however, generally form a correct judgment from the preceding history of the case; and when this cannot be ascertained, may often satisfy ourselves by observing the effects of pressure upon the epigastrium.

"A young man, an engraver by profession, and subject to dyspepsia, was suddenly attacked about an hour after his evening meal with an affection supposed to be apoplectic. When I arrived he was lying on his back, without motion, wholly unable to see or hear, and showing no sign of sensibility, even when pretty smartly shaken. He had

* North American Medical and Surgical Journal, No. XV.

not fainted; for the respiration and circulation continued; and the absence of stertor, with the natural condition of the pulse, was incompatible with the notion that the disease was apoplexy. Having learned that he had eaten cucumbers and whortleberries at his tea, and that he had afterwards complained of headach with some nausea, I had little doubt that the root of the evil would be found in the stomach. An emetic was accordingly administered, which, after a much longer interval than usual, operated freely, and brought away the undigested berries and cucumbers, which were undoubtedly the cause of irritation. Sensibility was now speedily restored; and after an attack of most violent spasm in the bowels, which was relieved by laudanum and castor oil, the patient recovered his usual health. He had never before been affected in a similar manner.

"Little more than a month has passed since I was requested to visit a gentleman said to be in a dying state. I found him apparently without sensation, his eyes open and turned up, his hands clenched, and his body alternately motionless, and agitated by sudden and universal tremors, which caused the bed to shake beneath him. He was a stranger, and there was no one present who could give me a history of his case. In order to explore into its nature I placed my hand upon his epigastrium; but scarcely had I touched the skin when he started up as though a bullet had been driven through him. Uncertain whether the coincidence might not be accidental, I repeated the experiment several times, and at each time the slightest pressure was sufficient to throw the whole frame into immediate and violent, though brief convulsions. Sufficient evidence was thus afforded of the seat of the disease, but not of the precise nature of the irritation. As his pulse was active I bled him freely, and immediately afterwards applied a large sinapism over the region of his stomach. Consciousness was so far restored a few minutes after the bleeding, that upon being asked in a loud voice if he felt sickness or pain in the stomach, he nodded in the affirmative. An injection of assafœtida was now administered; and under the united influence of this remedy and the mustard plaster, he revived to some knowledge of his situation, and was able to drink very freely of warm water, which I urged upon him. This soon produced the discharge of a considerable quantity of acid liquors from his stomach, and restored him for a time to complete consciousness. He now told me that he was subject to gout, of which he had recently had an attack in his foot, but had relieved himself by bathing the affected part with hot vinegar. The nature of the case was evident. While he was yet speaking, he was seized with a sudden spasm of the stomach, which threw him into his former state; and this alternation of consciousness and insensibility was repeated several times within the course of a few minutes, each return of pain being so severe as at first to throw the whole nervous system into violent agitation, and then to overwhelm it for a time in complete torpor. I now applied sinapisms to the feet, and gave a mixture of laudanum and the aromatic spirits of ammonia; and at the end of about four hours from the commencement of the attack, left my patient very greatly relieved. A dose of the compound tincture of rhubarb, with a proper regulation of the diet, was afterwards sufficient to complete the cure."

